# Variability of Microcirculation Detected by Blood Pulsation Imaging

**DOI:** 10.1371/journal.pone.0057117

**Published:** 2013-02-19

**Authors:** Alexei A. Kamshilin, Victor Teplov, Ervin Nippolainen, Serguei Miridonov, Rashid Giniatullin

**Affiliations:** 1 Department of Applied Physics, University of Eastern Finland, Kuopio, Finland; 2 Optics Department, Centro de Investigación Científica y de Educación Superior de Ensenada, Ensenada, Mexico; 3 Department of Neurobiology, A. I. Virtanen Institute, University of Eastern Finland, Kuopio, Finland; University Hospital of Würzburg, Germany

## Abstract

The non-invasive assessment of blood flow is invaluable for the diagnostic and monitoring treatment of numerous vascular and neurological diseases. We developed a non-invasive and non-contact method of blood pulsation imaging capable of visualizing and monitoring of the two-dimensional distribution of two key parameters of peripheral blood flow: the blood pulsation amplitude and blood pulsation phase. The method is based on the photoplethysmographic imaging in the reflection mode. In contrast with previous imaging systems we use new algorithm for data processing which allows two dimensional mapping of blood pulsations in large object's areas after every cardiac cycle. In our study we carried out the occlusion test of the arm and found (i) the extensive variability of 2D-distribution of blood pulsation amplitude from one cardiac cycle to another, and (ii) existence of the adjacent spots to which the blood is asynchronously supplied. These observations show that the method can be used for studying of the multicomponent regulation of peripheral blood circulation. The proposed technique is technologically simple and cost-effective, which makes it applicable for monitoring the peripheral microcirculation in clinical settings for example, in diagnostics or testing the efficiency of new medicines.

## Introduction

Imaging and monitoring of the variable blood flow in living tissues is of major importance in both biomedical research and clinical practice [1]–[6]. In the recent decades, several optical techniques have been developed for non-invasive and non-contact blood perfusion imaging for example Laser Doppler Flowmetry (LDF) [1]–[3] and the Laser Speckle Contrast techniques [4]–[6]. Photoplethysmography (PPG) is another optical technique which can be used for non-invasive detection of blood volume changes at the level of the microvascular bed [7]. This method was first developed in the 1930s [8], [9] and it has found various clinical applications. PPG technology has been incorporated into a wide range of medical devices for measuring oxygen saturation, blood pressure and cardiac output, assessing autonomic functions, and detecting peripheral vascular diseases. Implementation of PPG technique is very simple since it requires only two optoelectronic components: a light source to illuminate one part of the body and a photodetector to measure small temporal modulations in the light intensity [7]. Note that there is no need to use the coherent light in the PPG system in contrast with laser Doppler or laser speckle techniques: a simpler and cheaper light-emitting diode (LED) can be used instead. However, the wider application of PPG is limited by the fact that the typical PPG device can detect the blood flow in only a single point. In order to overcome this limitation, it was proposed that digital cameras could be utilized for the registration of blood volume changes in the tissue with the reflection-mode geometry [10], [11]. In this case, the digital camera simply records the video frames and the temporal modulation of each pixel value is associated with blood pulsations, assuming that the position of the studied area remains virtually unchanged during the cardiac period. However, the amplitude of this temporal modulation is very low compared to the time-averaged pixel value, and this requires that one resorts to image processing techniques to obtain a two-dimensional distribution of blood pulsation amplitude. Typically, the data processing for creation of PPG images [10], [11] is based on the Fast Fourier Transfer which is used for filtering out the output signal modulated at the heart-beat frequency and further estimation of blood volume pulsations.

A new method, called here blood pulsations imaging (BPI), was recently proposed for the production of PPG images from video recordings from living tissues using reflection geometry of PPG imaging [12]. The method is based on the lock-in amplification of every pixel of the recorded video frames. A reference function, required for synchronous detection of cardiovascular pulse waves in the PPG images, was formed from a large area of the same images. The main advantage of this approach is that after lock-in amplification, PPG images with increased signal-to-noise ratio (SNR) and high spatial resolution can be obtained [12]. However to achieve high enough SNR, the data averaging over several cardiac cycles (which takes 6–10 s) was required.

In this paper we describe a new algorithm of video-data processing in PPG imaging system, which allowed us to visualize two-dimensional distribution of blood pulsation amplitude and phase after every cardiac cycle. Combining high spatial and time resolution we are able to address the important question, how is the peripheral blood flow stable in time and uniform in space. The method was validated by reduced amplitude of blood pulsations in the course of an occlusion test and its subsequent restoration after pressure relief. To prove that our method concerns the blood flow, we demonstrated that the system response on a living subject is much higher than on an inanimate object, ensuring simultaneous measurements of both. These observations are evidence that the proposed system is capable of fast detection of changes in blood perfusion.

## Materials and Methods

### Image recording

**Figure 1:**
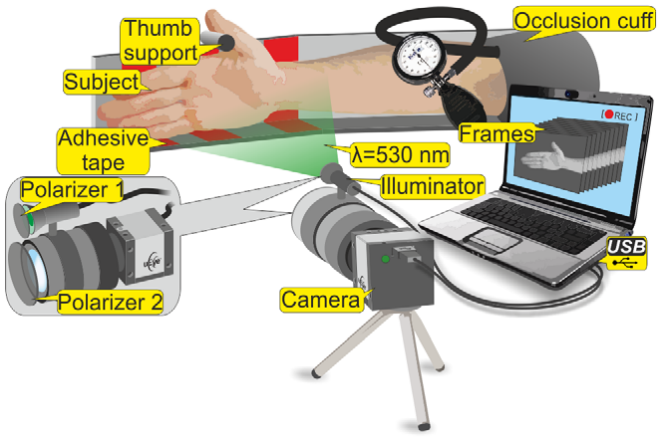
Experimental setup for blood perfusion imaging.

Blood pulsation imaging is a result of digital processing of video frames recorded as a conventional video by a digital camera. The setup for carrying out BPI experiments is shown in Fig 1. We illuminate the subject's hand (a palm and part of the wrist) by an illuminator which consists of 2 similar LEDs (model H2A3-530 of the Roithner LaserTechnik) operating at a wavelength of 530 nm. The LEDs were powered by a personal computer through USB ports which ensured not only portability of the system but also high stability of the subject's illumination. The optical power of both LEDs was ∼60 mW providing an illuminated area of 15×30 cm^2^ at a distance between the illuminated subject and the camera lens of 1 m. The illuminating light was linearly polarized by means of the film polarizer attached to the illuminator. The second polarizer was attached to the camera lens. The transmittance axes of the polarizers 1 and 2 were mutually orthogonal to reject the light reflected from the subject without any change in the polarization state. In contrast, the light with any modified polarization state is captured by the camera sensor. On one hand, a small fraction of the light reflected from the epidermal layers retains its polarization state. On the other hand, depolarization of the backscattered light occurs because of scattering events due to chromophores present in the tissue [13], [14]. By using crossed polarizers it was possible to separate a fraction of the diffusely scattered light from the surface reflections. The intensity of environmental illumination of the subject's area under the study was much lower than that provided by the LEDs. The light reflected from the subject's skin was collected into the camera to form a focused image of the subject area on the camera sensor.

We used a digital monochrome camera (8-bit model EO-1312 of the Edmund Optics) for capturing the light reflected from the subject. The illuminator was mounted above the camera lens (Canon TV-zoom 18-108) and the direction of illumination created a small angle with the optical axis of the lens. The camera was also connected to a personal computer though a USB port. Before starting the video recording, we used a preview window of the camera in the computer for targeting and focusing on the subject's area. Then by monitoring the online histogram of the pixel values, we adjusted either the intensity of the illuminating light or the exposure time of the camera in order to achieve an as high as possible dynamic range of pixels value but to avoid their saturation. After the adjustment was completed continuous video recording was started by saving one sequence of the frames in an internal memory of the digital camera and downloading these sequences to the computer in the uncompressed bitmap-image format. The parameters of the setup are listed in the Table 1.

**Table 1:** Parameters of the experimental setup of blood pulsations imaging.

Parameter	Value
Frame size	640×480 pixels
Frame rate	30 frames/s
Distance lens-subject	100 cm
Captured area	15 cm×30 cm
LED light wavelength	530 nm
LED light spectral bandwidth	40 nm
LED optical power	30 mW

### Ethics statement

This study was conducted in accordance with the ethical standards laid down in the 1964 Declaration of Helsinki. The study plan was approved on 08.02.2012 by the Research Ethics Committee, Hospital District of Northern Savo. The experiments were carried out with four different male subjects who are among the co-authors of this paper. All subjects gave their informed consent of participation in the experiment and in the publication of the results in the written form.

### Occlusion test

During the video recording, the subject with the occlusion cuff on his upper arm was asked to avoid any movement of his arm and keep breathing normally. In order to minimize possible movements the backside and lower part of the palm were fixed to the base by adhesive tape while the thumb was held tensed against the support. We started each experiment by recording the video frames of the relaxed subject when no air was inflated in the occlusion cuff for a period of 5 minutes. In the 6^th^ minute we increased the pressure in the cuff up to suprasystolic value of 180 mmHg. Inflation of the cuff was not instantaneous but it took about 20 s. This pressure was maintained for the next 2 minutes while continuously recording with the video camera. Thereafter, the air-valve of the cuff was opened, which led to an abrupt drop of the cuff pressure to the zero level. The recording of the video frames was continued for another 5 minutes. The whole sequence of the recorded frames lasted somewhat longer than 12 minutes and contained 23000 frames recorded at a frame rate of 30 fps. All the measurements were carried out in a dark room at an ambient temperature of 21–23 °C.

### Motion compensation

In an attempt to minimize the contribution of motion artifacts to BPI parameters during the prolonged (12 min) experiment we used several approaches. *First*, to stabilize the hand position with respect to the camera we used a stationary support shown in Fig 1. The subject's forearm leaned against the horizontal support, which eliminated muscle tension in the forearm and hence reduced the hand motion during the video recording. In addition, the back of the palm and its lower part were fixed to the support by adhesive tape while the thumb was located so that it was tensed against the small stick on the support. *Second*, we applied digital processing of the recorded frames for compensation of accidental movement of some parts of the arm (e.g. finger tremor). The custom software for this goal was implemented using the MATLAB® platform (of the Mathworks). This was achieved by splitting the whole frame into small regions and detecting the gradient of the pixel values within the subject's area in the frames sequence. Once the motion of any region was being detected, we stabilized exactly the region with moved gradient while the surrounding area remained stable and hence not modified. Note that our algorithm is designed to compensate for small displacements in a distance of few pixels. Larger displacements were prevented by the mechanical fixation of the whole arm. The motion compensation algorithm was executed before applying synchronous detection of cardiovascular pulse waves in PPG images.

### Reference function formation

**Figure 2:**
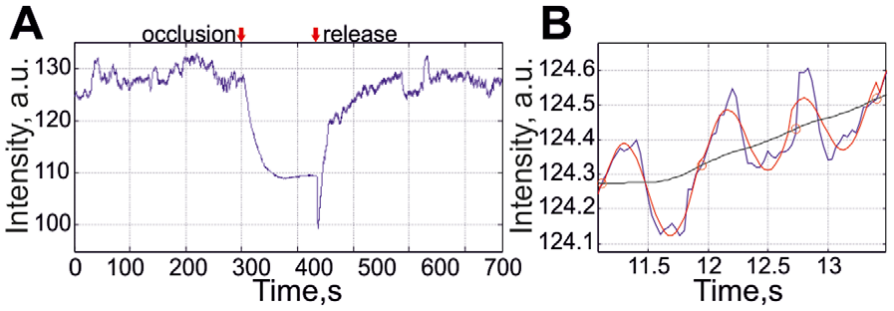
Time traces of raw PPG signal within the palm area. (A) An example of the signal during the complete occlusion test (about 12 min). (B) Zoomed part of the signal (blue line) with the temporal boundaries of each cardiac cycle (shown by red circles), which has been approximated to the harmonic reference signal (red dotted line). The black line in the part (B) is the raw PPG signal filtered by continuous averaging of the data over 30 frames.

In the mapping of the spatial distribution of blood pulsations, we applied the lock-in amplification technique which has been described in details in our recent paper [12]. Briefly, this technique is based on synchronous detection (lock-in amplification) of every pixel in the recorded video frames. The evaluation of spatial distribution of the blood pulsation amplitude (BPA) was executed as follows: first we generated the reference function which is essential for any lock-in amplification. Its formation started from averaging the pixel values over the whole area of the subject's palm. This averaging resulted in a single value per each recorded frame during the whole measurement. A typical example of the time-trace (raw PPG signal) of this value during the whole recorded video is shown in Fig 2a while a zoomed part of this time-trace (from 11 to 14 s) is shown in Fig 2b with higher temporal resolution. As one can see from Fig 2b, the raw PPG signal is modulated in time. This temporal modulation is caused by the intensity modulation of the backscattered light reflected from the subject's skin, which in its turn is mainly due to periodical blood volume modulation occurring synchronously with each heartbeat [7]. The observed modulation is associated with the heartbeat rate as in any PPG system [10]–[12].

It is well known that the heart rate of a subject is not constant but variable because of internal or external events influencing the vascular system of the subject [15]. In order to consider such variations, we estimated the duration of each cardiac cycle in the number of frames from the row PPG signal. To this end we first filtered the raw PPG signal by averaging the data over 30 sequential frames. This filtered signal is shown by the black curve in Fig 2b. Then we searched for the moments at which the raw PPG time-trace (blue curve in Fig 2b) crosses the black curve while having the positive derivation. These moments are shown by red circles in Fig 2b. Between these points we approximated the row PPG signal to a single period of the reference function. It was determined when the best correlation between the PPG and harmonic signals was obtained using the amplitude and the phase of the latter as free parameters for calculating the correlation [12]. An example of an approximation of the PPG signal by the harmonic function 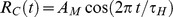 is shown in Fig 2b by the red curve. Here 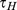 is the duration of a cardiac cycle and *A_M_* is the amplitude to be defined after normalization.

In the further visualization of the phase of blood pulsations, we generated the complex reference function *R*(*t*) with its real part equal to the synchronized *R_C_*(*t*) and an imaginary part equal to 90°-phase shifted *R_C_*(*t*), i.e. 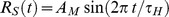. The reference function was normalized in such a way that 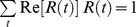 with the summation being conducted over all frames in the cardiac cycle under consideration. The function *R*(*t*) was further used for lock-in amplification of the recorded series of frames making it possible to calculate both the amplitude and the relative phase of each pixel-value oscillations for every cardiac cycle.

Note that formation of the reference function was impossible during the occlusion of blood vessels because there was no modulation of the raw PPG signal at the frequency close to the expected heart rate. Consequently, we could not estimate the duration of the cardiac cycle 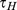 which is an important requirement for the formation of *R*(*t*). In this case, the reference function was generated using the mean 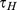 for the last minute before the occlusion event. After occlusion release, the modulation of the raw PPG signal at the heartbeat frequency was recovered and the function *R*(*t*) was generated in the same way as before the occlusion.

### Mapping blood pulsations

**Figure 3:**
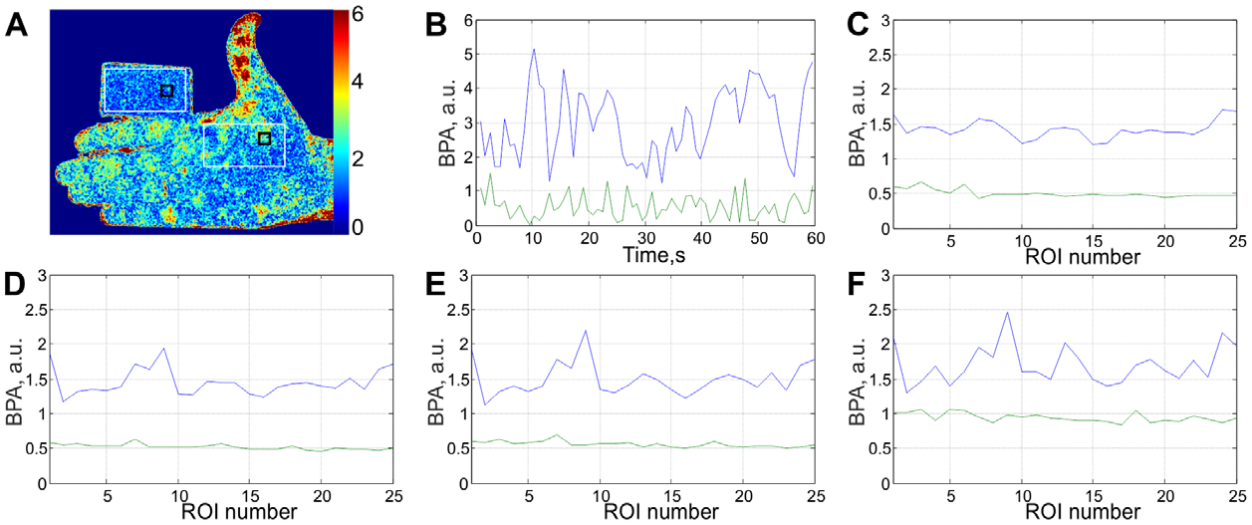
Estimation of the signal-to-noise ratio. (A) An example of 2D map of the blood pulsation amplitude for subject's arm and the cardboard; (B) Time-traces of the BPA averaged within 15×15 pixels regions shown by black rectangles in (A): the blue line for the arm and the green line for the cardboard; (C – F) Time-averaged BPA from randomly chosen regions within white rectangles sizing 30×30 (C), 15×15 (D), 8×8 (E) pixels, and for single pixel (F).

In order to visualize spatial distribution of blood pulsations, we calculated a correlation matrix 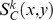 as 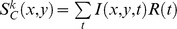 for each cardiac cycle numbered by *k*. Here *I*(*x*,*y*,*t*) is the value of a pixel with coordinates (*x*, *y*) of the image frame captured at the moment *t*, *R*(*t*) is the value of the reference function at the moment *t*. The correlation matrix 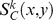 is complex and it has the same size as the recorded video frame. This matrix is the result of lock-in amplification of the pixel values varying in time synchronously with the generated reference function *R*(*t*). Therefore, the modulus of each pixel value of this matrix is proportional to the modulation amplitude of the light reflected from the respective point on the subject's skin. Since this modulation is caused by the blood pulsations related to the heart activity, the modulus of the correlation matrix, 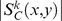, describes the spatial distribution of the blood volume pulsations after normalization with a value from the corresponding location of a time-averaged image matrix. This normalization is needed because the AC-component of the reflected light is a linear function of the intensity of the illuminating light, as was confirmed in additional experiments. The ratio of the AC-component to the mean level of the back-reflected light intensity represents the blood pulsation amplitude [16], [17]. Respectively, the argument of a complex value of the matrix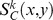 describes the spatial distribution of the blood-oscillations phase relative to the blood oscillations which are spatially averaged within the area selected for the formation of the reference function *R*(*t*). An example of the spatial distribution of BPA is shown in Fig 3a.

## Results

### Noise of the measuring system

Typically, precision estimates of any measurement value are assessed by the signal-to-noise ratio. In our experiment, we estimated the noise of the system by applying the same algorithm of the lock-in amplification to the measurements of an inanimate object. To this end we carried out an additional experiment using the same setup as shown in Fig 1 with a cardboard positioned adjacent to the subject's arm so, that the backscattered light from the subject and the cardboard were not correlated while both the cardboard and subject's arm were inside of the recorded frame. The intensity of the light reflected from the cardboard and the subject's arm were about the same. Note that in this measurement we did not perform the occlusion test.

The recording parameters were the same as shown in Table 1, while the recording time was 60 s. After downloading the recorded frames to the computer, we calculated the distribution of blood pulsations for each cardiac cycle by applying the synchronous detection technique described above. The typical distribution of the BPA is shown in Fig 3a.

We randomly selected two ROIs with size of 15×15 pixels (shown by black squares in Fig 3a) within the areas which are shown by white rectangles in Fig 3a, and then calculated the averaged BPA within each region. The averaging of the data from the correlation matrices was done by taking into consideration their phase, which resulted in a single complex value per each selected region per matrix. Then we calculated the modules of these averaged values for the whole set of the correlation matrices, which resulted in the time traces shown in Fig 3b. It can be clearly seen that the mean amplitude of the system response correlated with the heart beats calculated for the subject's arm is several times greater than that calculated for the cardboard (3.0 against 0.56). Moreover, variability of the response from the living subject is higher than from the inanimate object: the standard deviation is 1.0 and 0.35, respectively. The response from the inanimate object (cardboard) can be considered as an estimate of the noise level in the system.

In the further comparison of the system responses from a live subject and from an inanimate object, we arbitrarily chose 25 ROIs within the white rectangles shown in Fig 3a and studied the influence of the ROI size on the SNR, i.e. we did not change the positions of the centers of the chosen ROIs but varied their size. After the calculation of the time-traces for ROIs of different sizes, we plotted the time-averaged BPA as a function of the ROI number in Fig 3 (c–f). In all graphs, the mean BPA for inanimate object is shown by the green curve while for the living subject it is illustrated by the blue curve. It can be seen that in all cases (including the one-pixel ROI) the response from the subject is larger than that from the inanimate object. Note that data averaging within the ROI of larger size leads to diminishing of the response from the inanimate object because the phase of the pixel value of the correlation matrix is chaotically jumping from pixel to pixel. In contrast, decreasing of the averaged response from the live subject with an increased size of the ROI is much slower due to the phase correlation between adjacent pixels. However, the phase of blood pulsations is not evenly distributed over the subject area and not stable with time as it will be shown below, which are the main reasons why the signal declines with averaging within an ROI of larger size. We estimated that the SNR (the ratio of the system response for the live subject and inanimate object) is about the same for ROI of 30×30 and 8×8 pixels. The latter size of ROI (which corresponds to a physical size of 3×3 mm^2^ in the subject's palm with our particular imaging lens) was chosen for monitoring the BPA during the occlusion test because it provides the optimal spatial resolution.

### Variability of BPA maps

**Figure 4:**
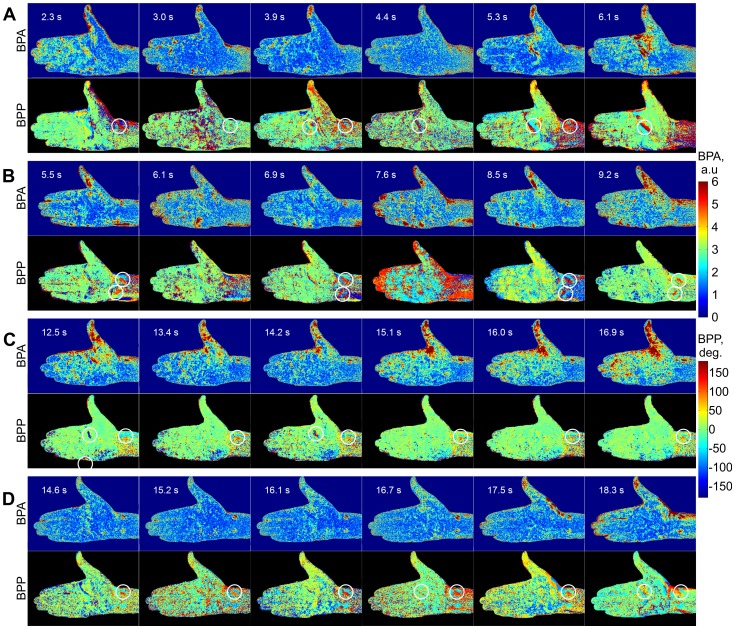
Spatial distributions of the blood pulsation amplitude (odd rows) and the relative phase of pulsations (even rows) calculated for four subjects (A–D) at sequential moments before occlusion of blood vessels. In the left corner of each map there is indicated the starting moment (in seconds) after which these maps are calculated for each cardiac cycle. The color scales on the right show the BPA (upper, in arbitrarily units) and the relative phase (lower, in degrees). White circles on phase maps show positions of asynchronous spots.

By applying the algorithm of the video-data processing described above, we were able to calculate the two-dimensional (2D) distributions of the blood pulsation amplitude (BPA) and their relative phase for each cardiac cycle during the whole experiment. Examples of these distributions calculated for 6 sequential cardiac cycles at the time before occlusion are shown in Fig 4 for every subject. From such a map, it is obvious that the blood pulsations are unevenly distributed over the subjects' arms and that these maps vary substantially from the subject to subject. There are clearly visible ‘hot’ spots in which the BPA is higher than in other areas of the arm. Note that the positions of these spots are more or less the same for each individual but their shape and relative amplitude are not stable but variable even when there are no external influences (like occlusion of blood vessels which was applied later on). From cycle to cycle, some of these spots disappear and then reappear again, which leads to a continuous changing in the locations of the hot spots. For all subjects, the largest BPA is observed at the area of the thumbs because of the thumb fixation. No hot spots were observed on the BPA maps during occlusion of blood vessels. 2D maps of the blood pulsation amplitude after occlusion release display the same features as before occlusion: they are extremely variable but the hot spots for each subject more frequently appears at the same positions indicating that they likely reflect the morphology of the vascular bed.

### Relative phase of blood pulsations

One important novelty is that, apart from the amplitude of blood pulsations (BPA), the present approach makes it possible to calculate the spatial distribution of the relative phase of blood pulsations (BPP). This parameter quantitatively indicates any delay in the blood supply reaching to different areas of the body and could contain physiologically important information of blood perfusion in these areas. We calculated the phase distribution with respect to the reference function used for the lock-in amplification at the heart-beat frequency. The 2D maps of the BPP are shown in each second row (with black background) of Fig 4 under the respective map of the BPA (with dark-blue background). It can be seen that the BPP is also non-uniformly distributed over the subject's arm. Moreover, the variability of the phase maps is higher than that of the amplitude maps. In spite of the instability in the phase maps, one can distinguish some adjacent areas at which the blood pulsates asynchronously. These areas are marked by white circles in Fig 4. Their physical size is about 1 cm. The phase difference in the blood pulsations in these adjacent areas is rather large, reaching 100° which means that the blood volume achieves its maximum in these adjacent areas in different instances with a time difference of about 200 ms. In most cases, these asynchronous spots coincide with the hot spots in the BPA maps.

### Monitoring of blood pulsations

**Figure 5:**
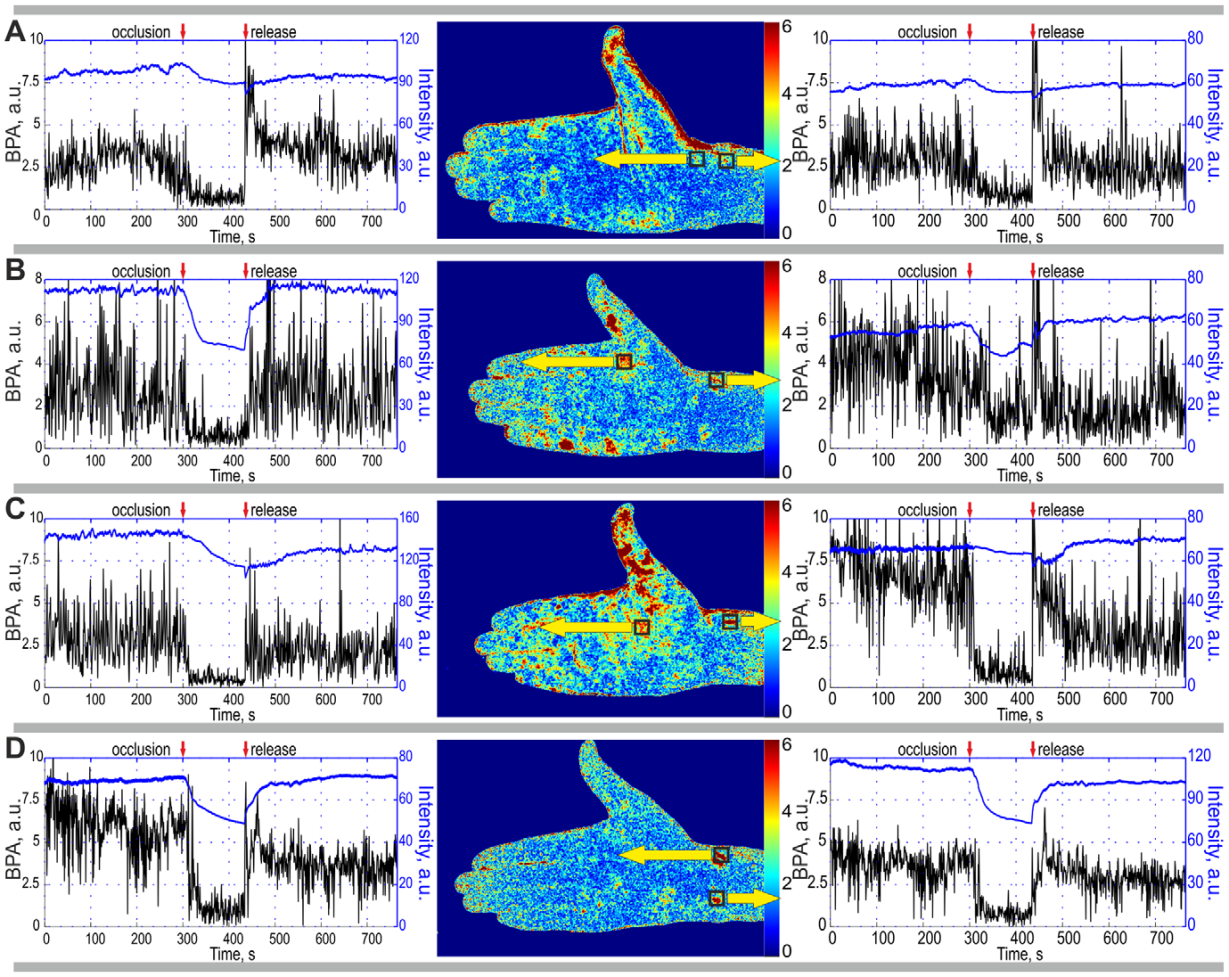
Time traces of the BPA (black curves) and the mean intensity of the back-reflected light (blue curves) calculated for four subjects by data averaging within selected ROI of 8×8 pixels. We placed the ROI in the ‘hot’ spots of 2D maps of the blood pulsation amplitude which are shown in the middle of each row. Yellow arrows indicate the ROI positions (which are shown in the BPA maps with the black squares) within which the respective graphs were calculated. Moments of blood vessels occlusion and its release are shown in every graph by red arrows.

By applying the synchronous detection technique to the whole number of the recorded frames we collected an array of the correlation matrices, the total number of which was equal to the number of cardiac cycles for an individual throughout the duration of the recording session. For different subjects, the number of cardiac cycles was different relating to their natural variations in cardiac activity. Then we averaged the complex values of the pixels for each correlation matrix 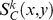 within the ROI of chosen size of 8×8 pixels. The reasons for the choice of this size have been discussed above. This averaging results in a single complex value per correlation matrix. By plotting the modulus of this mean value as a function of the matrix number *k* (which is unambiguously linked with the current time) we built up time-traces of the BPA shown in Fig 5. In order to ensure high SNR of the system, we placed the ROI at the position of hot spots which are marked by squares in 2D-maps of Fig 5.

By averaging the pixels value of the initial frames within the chosen ROI, it was possible to estimate the mean intensity of the light back-reflected from the respective subject's area. Time-traces of the mean intensity are shown by upper curves (blue lines) in the respective graphs of Fig 5. The lower curves (black and noisier) of the same graphs show the dynamics of the BPA which were calculated for the same ROI's of every cardiac cycle. It can be seen that the BPA declines in all subjects after beginning of the cuff inflation. The mean intensity also diminishes with approximately the same rate (about 20 s) as the cuff inflation. However in a few cases (right graphs in Fig 5a and Fig 5c), the diminishing of the mean intensity is not so marked.

The blood pulsation amplitude in any selected ROI is very variable, which is due to the extensive variability in the BPA maps shown in Fig 4. As can be see, this variability is different for different subjects and even for different ROI's of the same subject (the right graph against the left one of Fig 5a). The standard deviation (STD) of the BPA from its time-averaged value is much higher than that for an inanimate object. In the case of the second subject before occlusion (Fig 5b, right graph) STD was 2 for the subject but only of 0.4 for the inanimate object. If one compares two BPA time-traces of any of subjects, it is apparent that their variability is different. For example, for the first subject, the STD in the left ROI (Fig 5a, the left graph) is 2 times smaller than the corresponding STD in the right ROI (Fig 5a, the right graph) in spite of the similar time-averaged magnitude of the BPA. Since we applied the same data processing to the same recorded frames in the calculations of these pairs of time traces, we speculate that the differences in STD reflect features of the cardiovascular activity.

During the occlusion, both the mean BPA and its STD are comparable with respective parameters for the inanimate object in all subjects with the exception of one specific spot on the wrist of the second subject (the right graph in Fig 5b).

## Discussion

The proposed technique of blood pulsations imaging (BPI) made it possible to measure an alternating pulsative component of peripheral blood flow at heart-beat frequency. Due to high spatial and temporal resolution of our system, these measurements allowed mapping in large areas not only of the distribution of the amplitude of blood pulsations but also their relative phase. The main findings of this study are the observations of (i) the extensive variability present in both the BPA and BPP maps after each cardiac cycle, and (ii) the adjacent spots on the subjects' arms to which the blood is asynchronously supplied. We found that blood pulsations are not evenly distributed and they vary in time, even under normal conditions without any external influence being placed on the vascular system. Thus, our novel imaging system allowed us to detect positions of ‘hot’ and ‘asynchronous’ spots which have individual features representing a ‘pulsative signature’ of the individual.

### Methodological issues

We calculated the blood pulsation amplitude by processing the tissue images of the subject recorded in back-reflected light. In this study, the image processing was locked into the cardiac cycle but potentially the signal could be lock-in amplified at any biologically relevant frequency. Importantly, we minimized the following factors as sources of the system noise: (i) accidental mechanical movement of the whole hand, (ii) periodical modulation of the illuminating light power, (iii) random variations in the environmental illumination. Clearly observed modulation of the back-reflected light at the heart-beat frequency made it possible to conclude that the ratio of the temporally modulated amplitude of the pixel value to their time-averaged value represented the BPA.

### Comparison with LDF technique

Laser Doppler Flowmetry (LDF) is a popular technique to measure the peripheral blood flow [2], [3], [18]–[21]. It should be noted that the LDF and BPI techniques are visualizing different aspects of blood microcirculation. While the output signal of the LDF system is proportional to the velocity and concentration of the red blood cells, the signal of the BPI system is defined by the fractional blood volume proportional to the concentration of Hb. Therefore, these techniques provide us with the complementary information about functioning of the cardiovascular system. The amplitude of blood pulsations measured by the BPI system is somehow related with the blood perfusion measured by the LDF system. It could be assumed that the low perfusion is associated with low amplitude of blood pulsations and high perfusion can be achieved only when the pulsations of the blood is high because the blood is transported due to heart-driven hydraulic pulses [22]. However, the exact relationship between the perfusion and pulsations amplitude is still unknown. Both techniques are useful for mapping blood perfusion but the BPI is much simpler and it provides imaging of large areas of the subject with higher spatial resolution. In our experimental conditions, the light penetration depth is less than 1 mm [23], [24] because of the wavelength of 530 nm. Consequently the back-reflected light was presumably collected from superficial skin layers representing the microcirculation mainly in the capillary bed flow. Even though the LDF and BPI techniques are based on different principles, a similar uneven blood perfusion as to found in the current study has been recently reported by using an advanced LDF imaging system [3]. However, the BPI technique is more advantageous because it provides 2D-mapping of the blood pulsation phase and analyses largest areas of the subject.

### Variability of blood pulsations

We have observed that the 2D maps of blood volume pulsations (both the blood pulsation amplitude and relative phase) change after each cardiac cycle. One can speculate that the observed changes in the BPA/BPP maps are a direct consequence of well-known variability in the heart-rate [15]. However, changes of the maps were observed even though our algorithm took account of the heart-rate variability by estimating the duration of each cardiac cycle. Similar spontaneous fluctuations were observed [25] with conventional PPG system measuring blood pulsations in a single spot. We believe that such BPA/BPPs variability reflects changes in local vascular tone controlled by sympathetic and parasympathetic nerves. ‘Asynchronous’ spots most likely reflect the contribution of the endothelial, neurogenic, and myogenic components of microvascular flow control [19] or morphological peculiarities as an individual characteristic of the subject. Further studies of BPA/BPPs maps (especially their statistical properties) could provide a better understanding of the multi-component regulation of the peripheral blood circulation.

Since part of our algorithm of the video-data processing is devoted to the estimation of the actual period of the cardiac cycle, our system can be also used to study of the heart-rate variability. As an example we have listed in the Table 2 the mean value of the heart rate and its standard deviation for all four subjects who participated in the occlusion test.

**Table 2:** Mean heart rate and its standard deviation of four studied subjects.

Subject	Mean, beats/min	STD, beats/min
a	74	3.4
b	76	4.0
c	78	4.4
d	77	3.9

### Blood perfusion in the occlusion test

Since after occlusion of the blood vessels the heart-driven hydraulic pulses cannot be transmitted to the arm [22], one should expect significant diminishing of the BPA. Indeed, a dramatic drop in BPA was observed in most of our experiments with occlusion. This fact can be considered as evidence that the proposed system is truly responding to changes in blood perfusion. BPA maps calculated during the arterial occlusion with this reference function do not reveal any hot spots in all subjects: the blood pulsation amplitude does not differ from the system noise. We estimated the noise of our system by measuring the signal from an inanimate object. However, one exception was found in the wrist of the second subject (Fig 5B). In this spot, the mean BPA during occlusion is still about 2 times larger than the mean of the system response for the inanimate object. One possible explanation is that this hot spot represents a local myogenic activity which remains during the occlusion procedure. However, a more detailed study should be performed before drawing any conclusion about this phenomenon.

As seen in Fig 5 (blue curves), occlusion of blood vessels also leads to a diminishing of the mean light intensity back-scattered from the subject's arm. One explanation of this observation is as follows: since the blood pressure in the veins is lower than in arteries, the veins become blocked first soon the start of inflation of the cuff. This leads to some pumping blood into the arm by the arteries while the veins are closed. The increased blood volume results in greater absorption of the light [24] and consequently, in a diminished intensity of the back-scattered light.

### Blood reperfusion

Release of the cuff pressure leads to recovery of the blood supply to the arm but it typically takes about 1 minute to establish the same level of the blood volume as during the baseline. Figure 5 reveals that the mean intensity of the back-reflected light increases slowly after occlusion release. In contrast, the BPA recovers almost immediately in all subjects because there are no more obstacles against transmission of heart-driven hydraulic pulses to the arm after the cuff pressure release. During reperfusion in all subjects (Fig 5) we observed an overshoot when the BPA exceeds the baseline level. Note that the extent of this overshoot is different in the individual subjects. Moreover, it is also different in the different spots in the same subject. This phenomenon likely reflects the post occlusive reactive hyperemia in the vessels [18], [26]. We speculate that in the future, this phenomenon could be used to characterize the reactive functional properties of the vessels which are impaired in many vascular diseases including diabetes and atherosclerosis.

## Conclusions

In conclusion, we have developed a simple fast and non-invasive (and non-expensive) method for mapping of both the amplitude and relative phase of blood pulsations after each cardiac cycle, the parameters associated with the peripheral blood flow. It has allowed us to observe for the first time to our knowledge high variability of blood pulsations distribution which can be used for studying of the multicomponent regulation of peripheral blood circulation. Moreover, we have observed adjacent spots on the subjects' arms to which the blood is asynchronously supplied. The method of BPI is already suitable for measuring the human microcirculation in clinical settings including the diagnostic of vascular diseases and testing the efficacy of new medicines. Currently, our analysis is linked to the individual heart beat frequency but in the future, this method could be developed by improving the sensitivity, combining imaging obtained at different illuminating wavelengths, and locking the system into various biologically relevant (respiratory, myogenic or neurogenic) frequencies.
